# Determining the carbon transport time from Scots pine (*Pinus sylvestris* L.) needles to ectomycorrhizal sporocarps using the natural abundance carbon isotopic composition

**DOI:** 10.1093/treephys/tpaf130

**Published:** 2025-10-22

**Authors:** Lan Mo, Yann Salmon, Marco M Lehmann, Erik A Hobbie, Pauliina Schiestl-Aalto, Elina Sahlstedt, Yu Tang, Siiri Bienz, Giles H F Young, Katja T Rinne-Garmston

**Affiliations:** Stable Isotope Laboratory of Luke (SILL), Natural Resources Institute Finland, Latokartanonkaari 9, FI-00790, Helsinki, Finland; Institute for Atmospheric and Earth System Research (INAR)/Physics, Faculty of Science, University of Helsinki, P.O. Box 68, FI-00014, Helsinki, Finland; Institute for Atmospheric and Earth System Research (INAR)/Forest Sciences, Faculty of Agriculture and Forestry, University of Helsinki, P.O. Box 27, FI-00014, Helsinki, Finland; Forest Soils and Biogeochemistry, Swiss Federal Institute for Forest, Snow and Landscape Research WSL, Zürcherstrasse 111, Birmensdorf 8903, Switzerland; Earth Systems Research Center, University of New Hampshire, Durham, NH, 03824, USA; Institute for Atmospheric and Earth System Research (INAR)/Physics, Faculty of Science, University of Helsinki, P.O. Box 68, FI-00014, Helsinki, Finland; Stable Isotope Laboratory of Luke (SILL), Natural Resources Institute Finland, Latokartanonkaari 9, FI-00790, Helsinki, Finland; Stable Isotope Laboratory of Luke (SILL), Natural Resources Institute Finland, Latokartanonkaari 9, FI-00790, Helsinki, Finland; Institute for Atmospheric and Earth System Research (INAR)/Physics, Faculty of Science, University of Helsinki, P.O. Box 68, FI-00014, Helsinki, Finland; Department of Chemistry and Applied Biosciences, ETH Zurich, Vladimir-Prelog-Weg 3, Zurich 8093, Switzerland; Stable Isotope Laboratory of Luke (SILL), Natural Resources Institute Finland, Latokartanonkaari 9, FI-00790, Helsinki, Finland; Stable Isotope Laboratory of Luke (SILL), Natural Resources Institute Finland, Latokartanonkaari 9, FI-00790, Helsinki, Finland

**Keywords:** C allocation, C isotopes, intra-season δ_13_C dynamics, time lag, transport sugar, water-soluble carbohydrates

## Abstract

Ectomycorrhizal (ECM) fungi, as major carbon (C) sinks, are critical to plant–soil C cycling. Although C allocation between plants and ECM fungi has been studied extensively, C transport time, the key component of C cycling, remains poorly understood. To address this, we collected new needles (weekly), roots (monthly) and ECM fungi (sporocarps and hyphae) of three genera (*Cortinarius*, *Lactarius* and *Russula*) in a boreal Scots pine (*Pinus sylvestris* L.) forest in Finland. We analysed the natural abundance C isotope composition (δ^13^C) of sugars or organic matter and observed a strong vapour pressure deficit (VPD) signal in needle sucrose δ^13^C. We coupled VPD with the δ^13^C of water-soluble carbohydrates (WSC, δ^13^C_WSC_) in sporocarps to determine C transport times. We found *Lactarius* and *Russula*, with short hydrophilic mycelia that enable efficient solute uptake, had transport times of 6–13 days, peaking at 8 days. In contrast, *Cortinarius*, with extensive hydrophobic mycelia that limit water and solute movement, showed slower transport times of around 18 days. The different transport time is likely attributable to a more extensive mycelial network and potentially higher C demand in *Cortinarius* compared with *Lactarius* and *Russula.* The three genera also showed a marginally significant effect on δ^13^C_WSC_ in sporocarps (*P* = 0.06, analysis of covariant). This study highlights that natural abundance δ^13^C analysis offers a practical alternative to pulse-labelling for estimating C transport time in complex plant–fungal interactions, where the latter is difficult to implement. The longer transport time of *Cortinarius* compared with *Lactarius* and *Russula* is critical during periods of reduced photosynthesis, when limited C supply makes fast allocation essential for sustaining belowground metabolism. Slower transport may weaken its role and reduce forest productivity in boreal forests with short growing seasons. As global warming favours *Cortinarius*, its longer C transport time may impede soil C cycling and nutrient turnover.

## Graphical Abstract

**Graphical Abstract:**
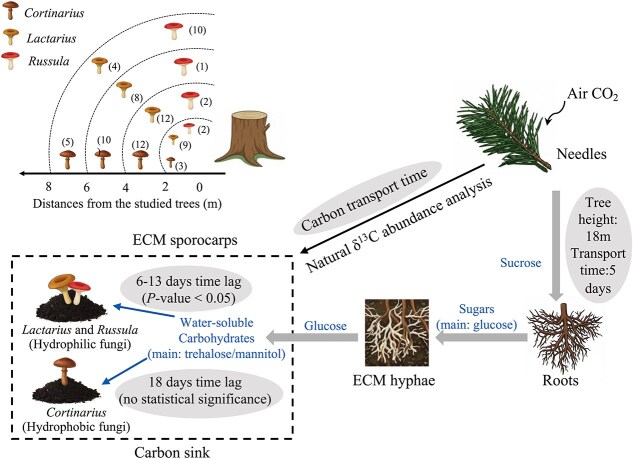

The figure presents the carbon (C) allocation from air CO_2_ to Scots pine needles, roots, ectomycorrhizal (ECM) hyphae and ECM sporocarps and estimates C transport times from pine needles to ECM sporocarps using natural δ^13^C abundance analysis. Photosynthetically fixed carbon is transported as sucrose from needles to roots, where it is converted to glucose prior to uptake by ECM hyphae. Two distinct fungal groups show different lag times from photosynthesis to sporocarps: *Lactarius* and *Russula*, classified as hydrophilic fungi, have a significant 6- to 13-day lag (*P* < 0.05), while *Cortinarius*, hydrophobic fungi, show a time lag of 18 days, but their statistical significance cannot be determined. Arrows indicate the transform and direction of C flow, and grey ellipses highlight the C trasnport times. The dashed box represents the C sink in the soil ecosystem. The upper-left panel shows the spatial distribution of ECM sporocarps relative to the studied Scots pine trees, indicating that different genera have distinct occurrence patterns with respect to distance. Numbers in brackets indicate the number of individual sporocarps collected at each distance.

## Introduction

Ectomycorrhizal (ECM) fungi play a critical role in belowground carbon (C) allocation and make an important contribution to the functioning of the forest soils (Clemmensen et al. 2013, Averill et al. 2014). A substantial amount of C (~9.07 Gt CO_2_e, carbon dioxide equivalent) is allocated annually to belowground ECM mycelium, equivalent to ~25% of current global annual CO_2_ emissions from fossil fuels, highlighting the importance of incorporating ECM fungi into global C and climate models (Hawkins et al. 2023). Ectomycorrhizal fungi form a mutualistic relationship with the roots of host trees and increase the efficiency of both parties in colonizing nutrient-limited environments (Nehls et al. 2010). In this symbiosis, trees provide photosynthetically fixed C and in return receive soil water and essential nutrients, such as phosphorus and nitrogen (N) (Nehls and Hampp 2000, Hobbie and Hobbie 2006). The C supplied by trees, up to 30% of the plant photosynthates, is essential for ECM fungal colonization of the soil, given their limited enzymatic capability to degrade complex soil carbohydrates (Colpaert and Van Tichelen 1996, Read and Perez-Moreno 2003, Martin et al. 2008). C input from trees is therefore an important factor in ECM fungal growth, as evidenced by the significant reduction in sporocarp production following host tree girdling (Högberg et al. 2001). Also, ECM fungal colonization is strongly related to the photosynthetic rate of host trees (Nara et al. 2003), and their sporocarps represent a major sink for host-derived C during the growth season (Vogt et al. 1982, Dahlberg et al. 1997). Ongoing climate changes, such as increasing temperature and rising atmospheric CO_2_ concentration, are likely to affect the role of ECM fungi in C cycling within terrestrial ecosystems (Pickles et al. 2012, Bennett and Classen 2020). Understanding the role of ECM sporocarps in forest C cycling and storage is therefore essential.

A key challenge in understanding C cycling between host trees and ECM fungi is determining the transfer time that links photosynthesis in tree leaves to C allocation in sporocarps (Moyano et al. 2008, Högberg et al. 2010, Kuzyakov and Gavrichkova 2010). The stable C isotope composition (δ^13^C) of organic matter reflects processes of C allocation and can improve assessments of C storage and cycling in soil (Kuzyakov and Gavrichkova 2010, Brüggemann et al. 2011). Previous studies have primarily employed two methodologies to investigate the C transport time in plant–soil systems (Phillips et al. 2012, Huang et al. 2024): ^13^CO_2_ pulse-labelling (Kuzyakov and Gavrichkova 2010) and natural abundance isotope analysis (Ekblad and Högberg 2001, Brüggemann et al. 2011, Hobbie et al. 2024). Using ^13^CO_2_ pulse-labelling, Högberg et al. (2008) observed a time lag of 4–7 days from needle photosynthesis to ECM hyphal respiration in a boreal Scots pine (*Pinus sylvestris L.*) forest. In the same experiment, peak ^13^C levels in sporocarps were detected at 6 and 16 days post-labelling (Högberg et al. 2010). At natural abundance levels, a lag of 7 days from photosynthesis to ECM sporocarps was reported for *P. sylvestris* in Sweden (Hobbie et al. 2024). These studies provided the foundation for assessing C transport times in ECM fungal ecosystems using δ^13^C as an indicator.

While ^13^CO_2_ pulse-labelling experiments are powerful tools for quantifying C allocation and velocity of C transfer by capturing instant δ^13^C variations (Dannoura et al. 2011, Wang et al. 2021), the method has limitations. They require artificial CO_2_ enrichment and leave detectable isotopic signals in the plant–soil system, which may interfere with long-term site integrity for future natural abundance isotopic measurements. Moreover, residual ^13^C signals following a pulse can persist in the system, making the approach less suitable for tracking C allocation at seasonal scales, as overlapping signals from repeat could complicate interpretation over time. Furthermore, in heterogenous root systems in mature forests, it is rarely feasible to sample root segments colonized by ECM fungi at the exact time of C transfer, due to the spatial complexity of the mycorrhizal network (Klein et al. 2023). However, when combined with metabolomic isotope analysis, pulse-labelling can provide detailed insights into C allocation processes by revealing the incorporation of recent assimilated C into specific metabolic compounds, such as amino acids and tricarboxylic acid intermediates in root tips (Rapaport et al. 2024). In addition, pulse-labelling can capture short-term C transport processes and time lag in a specific period, such as peak photosynthesis in late July (Epron et al. 2012). In comparison, although natural abundance analysis provides a weaker signal than pulse-labelling studies, it offers a more accessible approach for fieldwork. By relying on statistical analysis of δ^13^C changes, this method enables regular sampling over extended periods without disturbing long-term ecological monitoring sites. Notably, natural abundance δ^13^C in needle sugars not only reflects environmental conditions, such as solar radiation and air humidity, providing valuable insights into short-term physiological responses (Ekblad and Högberg 2001, Brendel et al. 2003, Tang et al. 2022, Hobbie et al. 2024), but also captures longer-term metabolic processes within plant organs. Sustained metabolic activity alters the isotopic composition of internal C pools, which is not easily accessible through pulse labelling, as the latter primarily tracks short-term C fluxes and offers limited information on cumulative turnover (Brüggemann et al. 2011). Furthermore, natural abundance enables the tracking of C transport time across tissues and over time, making it a practical and non-invasive tool for ecological studies in remote, resource-limited or spatially complex belowground ecosystems where pulse labelling is not feasible. However, the natural abundance analysis approach requires statistical analyses to interpret the more complex signal than that of pulse-labelling.

Despite advances in understanding the C transport time from canopy leaves to belowground components using natural abundance analysis (Knohl et al. 2005, Wingate et al. 2010, Hobbie et al. 2024), research specifically tracing the transport of sugars from needle photosynthates to genus-specific ECM sporocarps remains scarce. Sucrose is the primary product of photosynthesis and serves as the main transport form of recently assimilated C (Nehls et al. 2010). In the C transport pathway, sucrose is translocated via the phloem to the roots, which can receive half of a plant’s photosynthetically fixed C (Nehls et al. 2010). Upon reaching roots, sucrose is hydrolysed to its monosaccharides, i.e., glucose and fructose, and glucose is the key metabolic substrate for ECM fungal formation (Nehls et al. 2010, Ren et al. 2020). After uptake, ECM fungi further convert glucose into fungal-specific water-soluble carbohydrates (WSC) in sporocarps, mainly the disaccharide trehalose and the sugar alcohol mannitol (Nehls et al. 2010). At natural abundance, δ^13^C of needle sucrose provides a more accurate estimate of δ^13^C in newly assimilated C than total organic matter or bulk WSC (Tang et al. 2022). Compound-specific isotope analysis (CSIA) enables precise measurements of sucrose and glucose (δ^13^C_sucrose_ and δ^13^C_glucose_, respectively) in both needles and roots (Bowling et al. 2008, Rinne-Garmston et al. 2023, Tang et al. 2023). The δ^13^C of these fungi-specific WSC (hereafter δ^13^C_WSC_) in ECM sporocarps can be similarly measured. These approaches can facilitate the tracing of source C movement from needles through roots to ECM fungi. However, no study has yet used the natural abundance δ^13^C of soluble sugars and WSC to advance our understanding of the full C transport pathway from canopy to ECM fungi.

C in ECM sporocarps are mainly derived from photosynthetic C, with small contributions from soil-derived compounds (e.g., proteins) and host-derived metabolites (e.g., amino acids, nucleotides, fatty acids), potentially influencing δ^13^C_WSC_ of ECM sporocarps (Nehls et al. 2010, Hobbie et al. 2014). The mycelial exploration types of ECM fungi play a critical role in acquiring and sequestering the C sources from host species (e.g., trees) or soil organic matter (Hobbie and Agerer 2010). *Cortinarius* with extensive mycelial systems (medium-fringe exploration type) often require greater C inputs from their hosts to support the development and maintenance of large foraging networks. These fungi typically form hydrophobic, melanized cords that may restrict the movement of C and solutes within the mycorrhizal system and potentially delay C allocation to sporocarps (Agerer 2001). In contrast, *Lactarius* (contact exploration type) and *Russula* (medium-smooth exploration types) form shorter and more hydrophilic mycelia with limited soil foraging range, which are better suited for rapid exchange of WSC in root–fungal interactions (Ekblad et al. 2013, Suz et al. 2014). These morphological and physiological traits may influence C transfer efficiency, thereby influencing the C transport time to sporocarps (Lilleskov et al. 2011). Greater sequestration of ^13^C-enriched soil carbohydrates by *Cortinarius* may contribute to observed differences among taxa in δ^13^C relative to *Lactarius* and *Russula* (Hobbie et al. 2023). Consequently, it is necessary to investigate genus-specific effects when interpreting δ^13^C_WSC_ in ECM sporocarps.

To deepen our understanding of C allocation from host plants to ECM fungi, we tracked the transport of recently photosynthesized sugars from needles, via roots of *P. sylvestris*, to hyphae and ECM sporocarps during one growing season in Hyytiälä, Finland. Employing natural abundance δ^13^C analysis of WSC, complemented by CSIA of plant sugars, we estimated the time lag for C transport from needle photosynthesis to sporocarps formation. By detecting the time lag, we aim to demonstrate the applicability of natural abundance analysis in studying C allocation from the canopy to belowground. Our study also explored factors influencing δ^13^C_WSC_ in sporocarps and examined whether different ECM genera affect C transport time. We tested the following hypotheses: (i) natural abundance δ^13^C analysis can be used to detect the C transport time between needles and ECM sporocarps; (ii) the time lag can vary among different ECM genera, reflecting their unique physiological and ecological traits; (iii) ECM genera exert a significant influence on the δ^13^C_WSC_ in their sporocarps.

## Materials and methods

### Site description

The sampling site, Hyytiälä (61°51′N, 24°17′E, 170 m a.s.l.), is a managed boreal forest sown in 1962. The forest is dominated by Scots pine (*P. sylvestris*) mixed with Norway spruce (*Picea abies*) and birches (*Betula* spp*.*). The Scots pines were 57 years old in 2019. The soil is a Haplic Podzol on glacial till with some organic soil, it has a total depth of 5–150 cm, with an average depth of 5.4 cm for the organic layer (Hari et al. 2013, Machacova et al. 2019). The mean height of dominant trees was 18 m, and the stand density was 1177 trees ha^−1^ for trees over 1.3 m in 2016 (Aalto et al. 2019). The mean annual air temperature was 3.5 °C, and the mean annual precipitation was 711 mm for the period 1981–2020 (Pirinen et al. 2012).

### Environmental data

Environmental data for the study site were obtained from the Smart SMEAR AVAA portal (https://smear.avaa.csc.fi/), covering dates from 7 June to 11 October in 2019 (Abazajian et al. 2009, Aalto et al. 2023). Precipitation (mm) accumulated at 1-min intervals was recorded by a Vaisala FD12P weather sensor (Vaisala, Vantaa, Finland). Air temperature (T, °C) at 16.8 m height was measured hourly with a Pt100 temperature sensor (Peak Sensors Ltd, UK) located inside a ventilated custom-made radiation shield. Photosynthetically active radiation (PAR) (μmol m^−2^ s^−1^) was recorded at 35 m height every minute by a Li-190SZ quantum sensor (LI-Cor, Lincoln, NE, USA). Relative humidity (RH) at 16.8 m height was recorded every minute by the Rotronic MP102H RH sensor (Vaisala, Vantaa, Finland). Daytime means of PAR, air temperature and vapour pressure deficit (VPD) were calculated, as well as daily cumulative precipitation, defining daytime as the period 2 h after sunrise to 2 h before sunset.

### Sampling of Scots pine tree and ECM fungi materials

#### Tree tissue sampling

Sun-exposed current-year needles (0 N) from five mature Scots pine trees (mean height 18 m) were collected weekly from 7 June to 10 September in 2019 from 2 to 3 m below the top-most canopy level between 13:00 and 16:00 h on non-rainy days (a total of 13 sampling times). Fine roots were sampled from 5 to 15 cm soil depth using a 50 mm diameter soil corer from three randomly selected locations surrounding each sampled tree. This sampling procedure was carried out five times, twice in June and once per month from July to September.

Needles and root samples were placed in a cool box with ice blocks immediately after harvesting to minimize metabolic activity and micro-waved at 600 W for 1 min within 2 h to stop enzymatic and metabolic activities (Wanek et al. 2001). Then, Scots pine tree roots were selected from other roots under a light microscope after the wet soil samples were sieved. Subsequently, all samples were dried in a drying oven for 24 h at 60 °C and homogenized into a fine powder using a ceramic ball mill (FastPrep-24, MP Biomedicals, OH, USA).

#### Ectomycorrhizal fungi sampling

**Figure 1:**
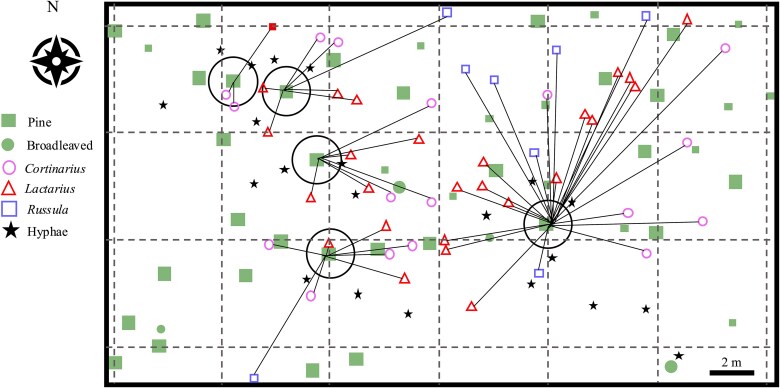
Location map of the five studied Scots pine trees (*Pinus sylvestris* L.) in Hyytiälä. Trees are shown as squares within open circles. Square size indicates two tree height classes (≈18 m and <18 m). Sampled sporocarps and associated hyphae are indicated by the corresponding symbols in the legend. Lines connect each sampled sporocarp to its nearest studied Scots pine tree, representing the assumed tree–fungus carbon source–sink relationships used for the carbon transport time analysis. Scale bar = 2 m.

We visited the site weekly from 19 July to 11 October to monitor ECM sporocarp presence within a 10 m radius of the host Scots pine trees, surveying the field site, Scots pine trees and ECM fungi sampling locations (Figure 1). We harvested all sporocarps of each genus present on each visit, ensuring no individual remained for potential resampling on later visits. This protocol ensured that the collected sporocarps were newly emerged and within a 7-day window of growth. All collected sporocarps and hyphae were promptly placed in a cold bag in the field and then microwaved at the Hyytiälä field station to stop all enzymatic activity (as above for the tree tissue materials). We then removed organic impurities with tweezers before further analysis. In the laboratory, half of the individual sporocarps were photographed under a microscope for genus identification and then stored in a freezer. Hyphae and the remaining half sporocarps were dried at 40 °C for 12 h for δ^13^C analysis and stored in a desiccator. After identifying collected ECM sporocarps, we found that only three dominant ECM fungal genera, *Lactarius*, *Russula* and *Cortinarius*, presented more than five replicates over the sampling season. Hence, we estimated the δ^13^C_WSC_ values and C transport time among these three ECM fungi genera.

### Sample preparation for δ^13^C isotope analysis

The dried tree tissues and ECM sporocarps were processed into a fine powder. Needles were milled in a ceramic ball mill (FastPrep-24, MP Biomedicals, OH, USA), and roots and sporocarps were processed in a steel-ball mill (MM 200, Retsch, Haan, Germany). All milling was applied for 30 s with a frequency of 25 s^−1^ and stored in plastic tubes. Due to the extremely small amount of hyphae materials, ~ 0.1 mg, milling into powder is a significant challenge. Hence, all hyphal materials were used for δ^13^C analysis of total organic matter (TOM, δ^13^C_TOM_).

### Extraction and purification of WSC

Extraction and purification of WSC from tree tissues (needles and roots) and ECM sporocarps were performed according to Wanek et al. (2001) and Rinne et al. (2012). Briefly, for the extraction, 60 mg of powdered tree tissues and 100 mg of powdered ECM sporocarp materials were placed in 2 mL reaction vials. The samples were suspended in 1.5 mL of deionized water and agitated using a vortex (ca 85 °C, VWR, PA, USA). Subsequently, the vials were placed in a water bath at 85 °C for 30 min, cooled down for 30 min and centrifuged at 10,000 × *g* for 2 min. The WSC supernatant of needle and roots was separated and purified using three types of sample treatment cartridges (Dionex OnGuard II H, A & P cartridges, Thermo Fisher Scientific) to remove amino acids, organic acids and phenolic compounds. Finally, the purified WSC were filtered through a 0.45 μm syringe filter and freeze-dried.

### Compound-specific δ^13^C analysis of soluble sugars in needles and roots

For needles and roots, the individual compounds in the purified WSC were analysed for δ^13^C using a Delta V Advantage isotope ratio mass spectrometer (IRMS) coupled with high-performance liquid chromatography via a Finnigan LC Isolink interface (Rinne et al. 2012). Four sugars or sugar alcohols with adequate concentration for CSIA (above 20 ng C μL^−1^) were consistently detected: sucrose, glucose, fructose and pinitol/myo-inositol.

Among these, only δ^13^C analysis of sucrose and glucose was used in our study. Fructose was excluded due to low concentrations throughout the root–fungus pathway, and pinitol was not considered as a key metabolite in mycorrhizal C exchange (Nehls et al. 2010). The needle samples underwent individual analysis at the Central Laboratory at the Swiss Federal Institute for Forest, Snow and Landscape Research (WSL lab, Birmensdorf, Switzerland), which provided the mean value and its standard deviations (SDs) for needle sucrose δ^13^C for each sampling day. In contrast, for root measurements, the five samples were pooled for each sampling date before measurements. Hence, the aggregation of these samples into a single composite for each date precluded the calculation of SDs for root sample δ^13^C values. A dilution series (20, 40, 60, 90, 120 and 180 ng C μL^−1^) of external compound-matched standard solutions was analysed between every 10 samples to calculate sample concentrations and correct δ^13^C values.

Measurement precision determined from multiple analyses of a quality control material was 0.2‰ (SD).


(1) \begin{equation*} {\delta}^{13}C=\left(\frac{R_{sample}}{R_{standard}}-1\right)\times 1000 \end{equation*}

In Eq. (1) , *R_sample_* and *R_standard_* are the ^13^C/^12^C ratio in the sample and the standard, respectively. The reference material for *δ*^13^*C* calculations is Vienna-Pee Dee Belemnite.

### δ^13^C analysis of WSC and TOM of ECM fungi

Aliquots of WSC from sporocarps were pipetted into individual tin capsules (5 × 9 mm, Säntis, Teufen, Switzerland), freeze-dried and wrapped. The TOM of sporocarps and hyphae were weighed 0.88 mg and 0.1 mg, respectively, into tin capsules (3.3 × 5 mm, Säntis, Teufen, Switzerland) at WSL. δ^13^C_WSC_ and δ^13^C_TOM_ in sporocarps samples and δ^13^C_TOM_ in hyphae were determined at WSL with an Elemental Analyzer coupled via a Conflo III reference unit to an IRMS Delta^XP^Plus (Thermo Fisher, Bremen, Germany). Measurement precision determined from multiple analyses of a quality control material was 0.1‰ (SDs).

Our study relied on δ^13^C_TOM_ for hyphae, particularly in the context of ^13^C translocation in sugar compounds in plant–fungi interaction, prompting us to evaluate its effectiveness. We conducted Pearson’s correlation analysis (Pearson’s *r* = 0.75, *P*-value < 0.001) and paired *t*-tests (*t*-test, *P*-value > 0.05) for the sporocarp data, revealing a high correlation and no significant differences between δ^13^C_WSC_ and δ^13^C_TOM_. These findings suggested that δ^13^C_TOM_ can serve as a viable proxy for δ^13^C_WSC_.

### Carbon transport time estimation

#### Environmental proxy for δ^13^C of needle sucrsoe

The temporal resolution of our δ^13^C analysis of needle sucrose (δ^13^C_sucrose_) was constrained by the limited availability of newly formed needles and the analytical complexity of CSIA, which required extensive sample preparation and long run times. As a result, analyses of C transport dynamics based on our discrete, low-frequency δ^13^C_sucrose_ dataset are statistically challenging. To address this issue, we established relationships between needle δ^13^C_sucrose_ and continuous data of local environmental variables (VPD, PAR and T) that regulate canopy stomatal conductance and photosynthesis and thereby influence δ^13^C signatures in needles (Bowling et al. 2002). These local environmental variables can serve as proxies for recent photosynthetic activity and have been used to infer C transport times (Ekblad and Högberg 2001, McDowell et al. 2004). This analysis enabled us to develop a transfer function between δ^13^C_sucrose_ and environmental data, and thus facilitate model δ^13^C_sucrose_ at high resolution over the entire growing season. The key aim of this approach was to fill the temporal data gaps in δ^13^C_sucrose_ data and facilitate a more thorough statistical exploration of the C transport time between leaf photosynthesis and sporocarp formation.

We assumed that leaf δ^13^C_sucrose_ accurately records changes in leaf gas exchange, which is driven by environmental variables such as VPD, RH and T and PAR (Rinne et al. 2015, Tang et al. 2023). We accordingly calculated Pearson’s correlations between δ^13^C_sucrose_ in needles and these three environmental variables. This was done to identify the most relevant environmental proxy for time lag estimation by selecting the signal with the strongest correlation coefficient (*P* < 0.05). First, we calculated the average δ^13^C_sucrose_ value for each needle sampling date to create a time series dataset of δ^13^C_sucrose_ in needles. Subsequently, due to the carry-over effect in the leaf sugar pool, which mixes new and old assimilates over the past several days (Streit et al. 2012, Leppä et al. 2022), we applied the time-integrated environmental signals to reflect the integrated δ^13^C_sucrose_ according to the methodology of Tang et al. (2023) (Eq. (2), below). These signals contained integrated environmental information from several days prior to the needle sampling dates with variable weights.


(2) \begin{equation*} {x}_t^{\ast }=\frac{\left({\sum}_{i=0}^n{\lambda}^i\times{x}_{t-i}\right)}{\sum_{i=0}^n{\lambda}^i} \end{equation*}

where ${x}_t^{\ast }$ is the integrated variable on day *t* (i.e., VPD, air temperature and PAR); *i* is the number of days prior to day *t*; *n* is the number of days calculated, which varies from 0 to 12 days at an interval of 1 day; λ is the previous day’s weight, which varies from 0 to 1 at an interval of 0.1; ${x}_{t-i}$ is the variable at day *t−i*; λ defines the percentage of δ^13^C_sucrose_ needles on the current day that is reserved for the following day. Using Eq. (2), we calculated time-integrated environmental variables with variable λ (0 to 1) and time spans (0 to 12 days).

#### δ^13^C_WSC_ datasets of ECM sporocarps

We derived temporal δ^13^C_WSC_ datasets for three ECM fungi genera and calculated the average δ^13^C_WSC_ for each genus based on their sampling dates. However, the limited number of sporocarps for *Russula* (*n* < 3) on several dates and the occasional absence of *Lactarius* and *Cortinarius* posed statistical challenges in estimating time lags at each genus level (Table S1 available as Supplementary Data at *Tree Physiology* Online). To overcome this limitation, we combined δ^13^C_WSC_ data of *Lactarius* and *Russula* and averaged their values for each sampling date, as both are hydrophilic (Hi) fungi (Lilleskov et al. 2011) and did not differ in their δ^13^C_WSC_ values (independent *t*-test, *P*-value > 0.05). This resulted in the creation of the Hi group δ^13^C_WSC_ dataset. *Cortinarius*, the hydrophobic (Ho) fungi (Lilleskov et al. 2011), remained as a separate δ^13^C_WSC_ dataset. This integration of the Hi fungal dataset enhanced the reliability and precision of C transport time estimations.

#### Carbon transport time estimation

We estimated the possible C transport time from the canopy to ECM sporocarps at between 0 and 30 days. Accordingly, we measured the time-integrated environmental proxy over a continuous period of 30 days prior to the sporocarps sampling dates and then applied Pearson’s correlation to determine the time lag by assessing the relationship with intra-season δ^13^C_WSC_ datasets of the Hi group and Ho fungi (*Cortinarius*). However, the environmental proxy datasets had high levels of autocorrelation, resulting from the integration of environmental signals in the needle photosynthates over previous days with variable weights. This phenomenon manifested in numerous significant findings (*P* < 0.05), potentially leading to false-positive time lags. To account for this issue, we calculated the *effective n* (N_cor_) using the method of Dawdy et al. (1964), as denoted in Eq. (3).


(3) \begin{equation*} {N}_{cor}=N\frac{1-{r}_{1,x}{r}_{1,y}}{1+{r}_{1,x}{r}_{1,y}a} \end{equation*}

*N* is the sample size, *N_cor_* is the effective sample size and *r*_1_ is the first-order autocorrelation coefficient. *x* is the intra-season δ^13^C_WSC_ values of the Hi group or *Cortinarius*, and *y* are the proxy with corresponding time lags, respectively. *r*_1,*x*_ and *r*_1,*y*_ are the first-order Pearson’s autocorrelation coefficients of time series *x* and *y*.

This technique involves reducing the sample size (N) to obtain an effective sample size (*effective n*) that aligns with the autocorrelation presented in the datasets under analysis. Following this adjustment, we recalculated the *P* using the *effective n* through Free Statistics Calculators (https://www.danielsoper.com/statcalc). Ultimately, when the correlation coefficients were significant (*P* < 0.05), we identified the C transport time from needles to the Hi group and to *Cortinarius*.

### Statistical analysis

We assessed the effects of ECM fungal genera (*Cortinarius, Lactarius* and *Russula*), sampling the day of year (DOY) and the distance between each sporocarp and its nearest studied tree (graded 1–8; e.g., < 1 m as 1, 1–2 m as 2 and so on) on δ^13^C_WSC_ of sporocarps using analysis of covariant (ANCOVA). Notably, because the actual host tree for each sporocarps could not be determined, we used the nearest studied tree as the most likely host. The distance was recorded in broad classes rather than as exact measurements and was therefore treated as a categorical variable in the analyses. Due to limited genera on hydrophobicity classification, with only one genus, *Cortinarius*, categorized as hydrophobic (Ho) and two, *Lactarius* and *Russula*, as hydrophilic (Hi), we excluded hydrophobicity as a factor from our analysis. In this model, genera and DOY were treated as fixed factors, and distance as a covariate, with all interaction terms included. Due to unbalanced sample sizes and the presence of missing combinations in higher-order interactions (e.g., genera × DOY × distance), we used Type II sum of squares to evaluate the significance of each term. All significances were assessed based on the *F*-statistics. The analysis was conducted using the Anova () function from the car package in R (version 4.3.2, 2023), after fitting the model with lm ().

## Results

### Environmental conditions

**Figure 2:**
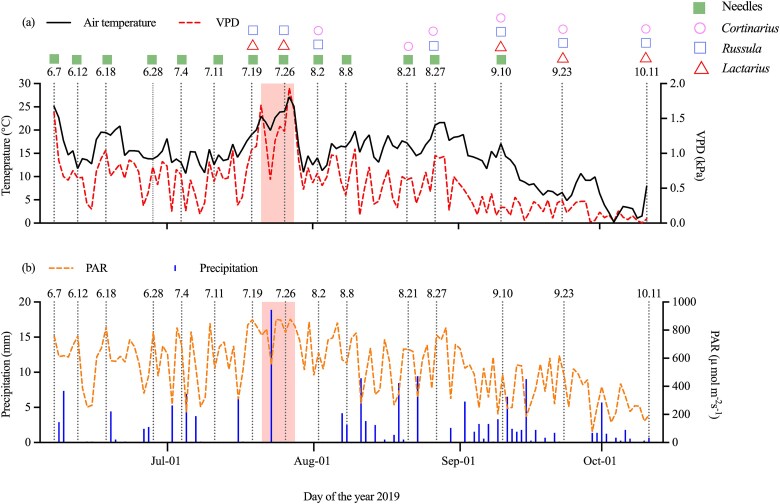
Daytime environmental conditions during the growing season of Scots pine (*Pinus sylvestris L.*) needles and fungal sporocarps from 7 June to 11 October 2019. (a) Air temperature (T) and vapour pressure deficit (VPD), (b) precipitation and photosynthetically active radiation (PAR) from 2 h after sunrise to 2 h before sunset. Vertical dotted lines indicate all sampling dates. The shaded areas indicate a high VPD period between the two peaks. Sampling dates of needles and *Cortinarius*, *Russula* and *Lactarius* are shown at the top of the panel.

Over the study period from 7 June to 11 October, air temperature (T) varied from 0.2 °C to 27.2 °C with an average value of 14.2 °C (Figure 2a). The VPD reached high values at 1.9 and 1.7 kPa, both at the beginning of June and at the end of July, respectively, and decreased to 0 kPa by 11 October (Figure 2a), with a mean value of 0.6 kPa over the growing season. Daytime PAR values remained consistently high in late July and then gradually decreased towards October, with considerable fluctuations over the period. Precipitation was intermittent but generally uniform across the study period, with increased frequency during September (Figure 2b). Peak precipitation of 18.9 mm was on 21 July.

### δ^13^C pattern in needles, roots and hyphae

**Figure 3:**
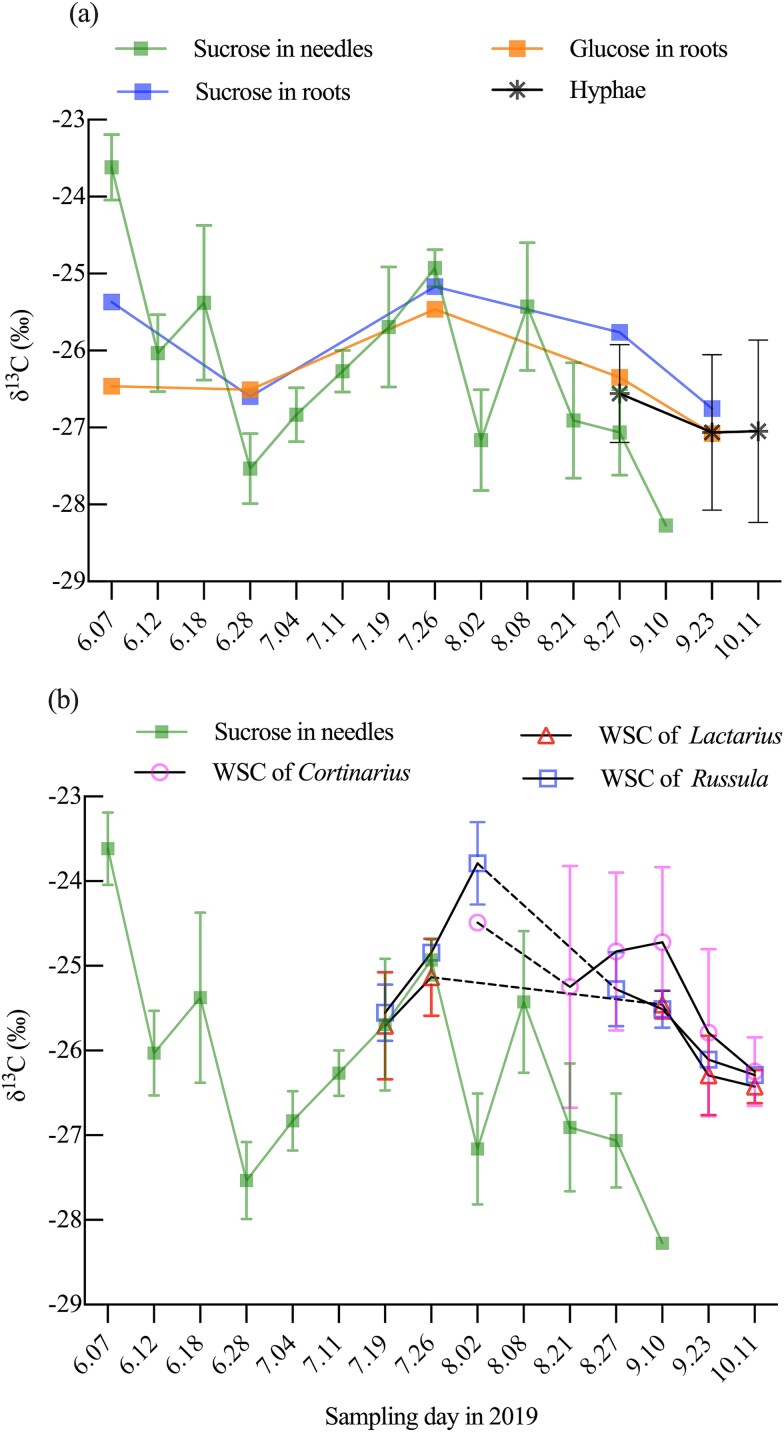
Comparison of intra-seasonal δ^13^C dynamics in Scots pine (*Pinus sylvestris L.*) tree tissues and ectomycorrhizal (ECM) fungi species. (a) δ^13^C of sucrose in Scots pine needles, sucrose and glucose in Scots pine roots, and total organic matter of hyphae. (b) δ^13^C of sucrose in Scots pine needles and water-soluble carbohydrates (WSC) in sporocarps of three ECM species. For clarity, dashed lines indicate periods during which no sporocarps were collected. Mean values and standard deviation are shown (roots were pooled; needles (*n*) = 5; hyphae (*n*) = 6–13; sporocarps: *Cortinarius* (*n*) = 1–9, *Russula* (*n*) = 1–5, *Lactarius* (*n*) = 3–14).

The maximum δ^13^C_sucrose_ in needles was on 7 June, followed by the second-highest value on 26 July (Figure 3a). These peaks were interspersed with a temporary decline, followed by a gradual decrease. In comparison, δ^13^C_sucrose_ in roots, which were collected less frequently, were also higher on these same dates relative to other sampling occasions (Figure 3a). The δ^13^C_glucose_ in roots generally followed the δ^13^C_sucrose_ trends in roots and peaked on 26 July.

There was no consistent offset in the δ^13^C_sucrose_ values between needles and roots. For example, on 7 June, δ^13^C_sucrose_ in needles was higher than in roots by 1.75‰, while on 28 June and 27 August, roots had higher δ^13^C_sucrose_ than needles by 0.94‰ and 1.30‰, respectively. On 26 July, δ^13^C_sucrose_ in needles shared similar values with roots. On average, the δ^13^C_sucrose_ in needles (−26.1 ± 1.2‰) closely approximated that in roots (−25.9 ± 0.6‰). Overall, the average of δ^13^C_TOM_ in hyphae was −27.0 ± 1.0‰ (Table 1). On 27 August and 23 September, when hyphal δ^13^C measurements were available alongside plant sugar δ^13^C data, δ^13^C_glucose_ values in roots were close to the average δ^13^C_TOM_ in hyphae (Figure 3a).

**Table 1:** δ^13^C values of water-soluble carbohydrates (WSC) in three ectomycorrhizal (ECM) fungal genera and of total organic matter (TOM) of hyphae during the fungi growth period between 19 July and 11 October 2019 (mean ± SD). Hydrophobicity: Hi = hydrophilic, Ho = hydrophobic. Different letters after numbers indicate statistical difference at *P* < 0.05.

ECM fungi (*n*)	Hydrophobicity type	δ^13^C (TOM, ‰)	δ^13^C (WSC, ‰)
*Cortinarius* (30)	Ho	−25.1 ± 1.0	−25.2 ± 1.0
*Russula* (15)	Hi	−24.9 ± 0.7	−25.3 ± 0.8
*Lactarius* (33)	Hi	−25.6 ± 0.5	−25.9 ± 0.7
Average fungi (78)	–	−25.3 ± 0.8 a	−25.5 ± 0.9
Hyphae (31)	–	−27.0 ± 1.0 b	–

### δ^13^C patterns in ECM sporocarps

The availability of sporocarps across the sampling dates was not evenly distributed: the three studied genera appeared together only from 10 September to 11 October; the Hi group (*Lactarius* and *Russula*) was absent on 21 August, and *Cortinarius* was not detected during all of July (Figure 3b). Notably, we collected only one specimen of *Cortinarius* and two of the Hi group on 2 August, and there were only two specimens of *Cortinarius* on 21 August (Table S1 available as Supplementary Data at *Tree Physiology* Online).

Considering the SDs of δ^13^C_WSC_, we observed an increasing trend in δ^13^C_WSC_ of *Russula* from 19 July (−25.6 ± 0.3‰) to their peak on 2 August (−23.8 ± 0.5‰), followed by a declining trend until 11 October. The δ^13^C_WSC_ of *Lactarius* were similar to those of *Russula* on each common sampling day (Figure 3b). From 27 August to 11 October, when multiple samples of *Cortinarius* were collected (*n* > 3), the δ^13^C_WSC_ values for specific sampling days had high SDs, with a general trend towards lower values from 10 September onwards, approaching a minimum of −26.2‰ ± 0.4‰ (*n* = 5). These large SDs suggested that this trend should be interpreted with caution. Overall, the average δ^13^C_WSC_ and δ^13^C_TOM_ values were similar across the three fungal genera (Table 1).

### Factors driving δ^13^C values of ECM sporocarps

The ANCOVA results (Table 2) showed that DOY was the single significant factor influencing δ^13^C_WSC_ values of sporocarps (*F* = 9.89, *P* < 0.001), indicating substantial temporal variation in δ^13^C_WSC_ across the sampling period from July to October. The effect of ECM genera was not a significant factor, but it approached statistical significance (*P* = 0.06), suggesting a potential influence of genera on δ^13^C_WSC_ of ECM sporocarps. Moreover, the distance showed no significant effect, while all interactions involving distance were significant, suggesting that its influence on δ^13^C_WSC_ in sporocarps was genus-dependent, with some genera exhibiting stronger responses, implying that distance may modulate genus-specific patterns of C allocation.

**Table 2:** Results of Type II ANCOVA analysing factors, including sampling date (DOY), ECM fungi genera (genus) as fixed factors and classification of distances to the nearest studied tree distance (distance) as a covariant, affecting δ^13^C values of water-soluble carbohydrates (δ^13^C_WSC_) in 78 sporocarps from three ectomycorrhizal (ECM) fungal genera. Significant *P*-values are given in bold (*P* < 0.05). N.S. represents no significant difference.

Variables	*df*	*F*-value	*P-*value	Interactions	*df*	*F*-value	*P-*value
Genus	2	2.96	0.06	DOY × genus	3	0.08	N.S.
DOY	7	9.89	**<0.001**	Distance × genus	4	3.46	**0.02**
Distance	7	2.13	N.S.	DOY × distance	10	1.43	N.S.

### Utilizing VPD as the environmental proxy for needle δ^13^C_sucrose_

**Figure 4:**
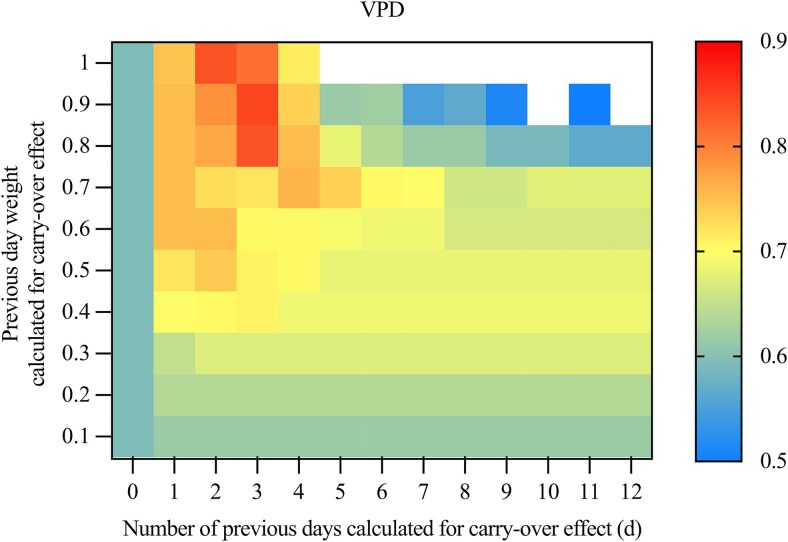
The environmental proxy was shown to estimate C transport time, with the integration of the vapour pressure deficit (VPD) signal in δ^13^C of needle sucrose in Scots pine (*Pinus sylvestris L.*) during the 2019 growing season at Hyytiälä. The optimal model integrated the environmental proxy signal for the current day (d) and past 3 days (d, d–1, d–2, d–3) and a fraction of the previous day’s weight of 0.9 (0.9^0^, 0.9^1^, 0.9^2^, 0.9^3^) using Eq. (2). Pearson’s correlation (*r*) coefficient is indicated by the numerical value shown for each model configuration, ranging from low to high values (with the highest values obtained using a 0.9 daily weight at three previous days). Only significant results (*r* > 0.5, *P* < 0.05) are presented.

**Figure 5:**
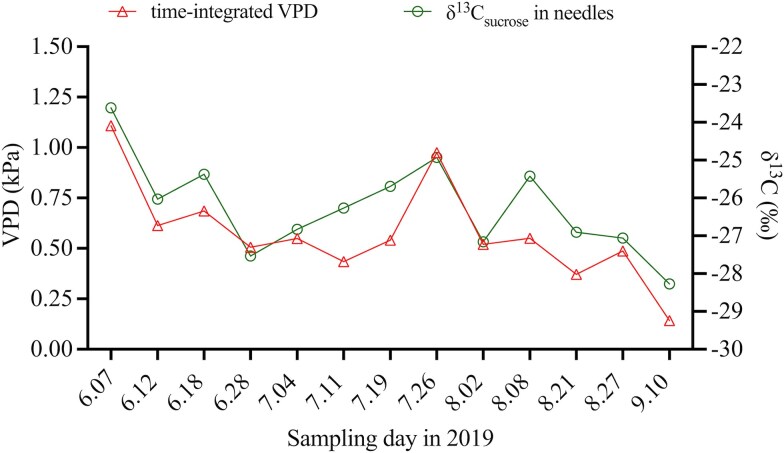
Time series of δ^13^C_sucrose_ in needles (circles) and time-integrated vapour pressure deficit (VPD, triangles) during the 2019 growing season. The VPD values were calculated using a carry-over model with a daily weighting factor of 0.9 over the preceding 3 days, following the approach of Tang et al. (2023).

Among the environmental variable measurements, δ^13^C_sucrose_ in needles had a higher correlation with VPD (Pearson’s *r* > 0.80, *P* < 0.05) than with RH, air temperature and PAR (Figure 4 and Figure S1 available as Supplementary Data at *Tree Physiology* Online). We therefore selected VPD as the environmental proxy for δ^13^C_sucrose_ in needles, similar to Hobbie et al. (2024), who used time-integrated solar radiation to estimate C transfer lags between trees and sporocarps. Furthermore, the strength of the correlation between δ^13^C_sucrose_ in needles and VPD was improved when we considered the carry-over effect of sucrose in needles. The relationship was most notable (Pearson’s *r* = 0.85, *P* < 0.001) when incorporating a fraction of the previous day’s signal from day 0 to 3 (over a 4-day period), and a previous day’s weight of 0.9, i.e., a time-integrated VPD (Figure 4). This can be described by the optimal model: ${x}_t^{\ast }=\frac{\left({\sum}_{i=0}^3{0.9}^i\times{x}_{t-i}\right)}{\sum_{i=0}^3{0.9}^i}$. Using this optimal model, time series dataset of δ^13^C_sucrose_ in needles exhibited a pattern that closely followed the time-integrated VPD variations, with higher δ^13^C_sucrose_ in needles corresponding to higher VPD values (Figure 5). These findings suggested that this time-integrated VPD can effectively model and reflect the sucrose composition in the needle sugar pool by linking the temporal resolution of VPD into δ^13^C_sucrose_ through changes in stomatal conductance and isotopic discrimination_,_ confirming the reliability of this approach. In our dataset, VPD was strongly collinear with air temperature, RH and PAR, and model comparison showed that VPD explained the observed δ^13^C variations in sporocarps more robustly (Figure S1 available as Supplementary Data at *Tree Physiology* Online). We therefore selected time-integrated VPD as a predictor variable for δ^13^C_sucrose_ in needles.

### Estimation of carbon transport time from Scots pine needles to ECM sporocarps

**Figure 6:**
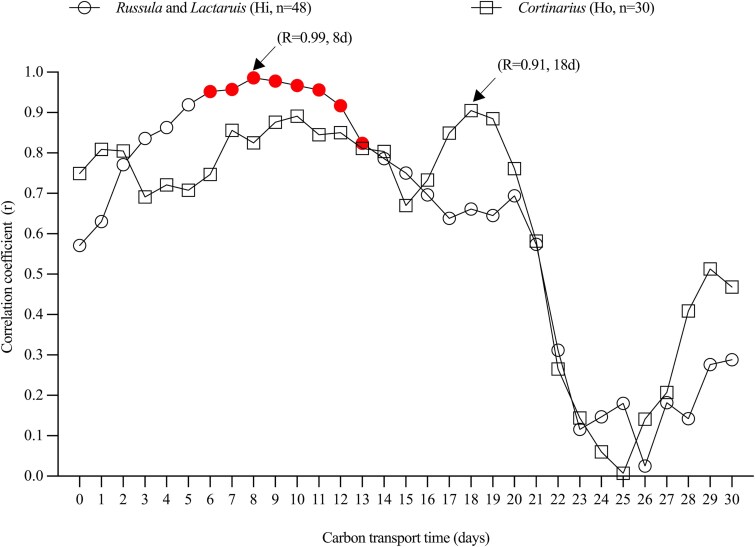
Coefficients of correlation between time-integrated vapour pressure deficit (VPD), based on the optimal model from Figure 4, and δ^13^C of water-soluble carbohydrates (WSC, δ^13^C_WSC_) in ectomycorrhizal (ECM) sporocarps species as a function of C transport time (the time lag between photosynthates in Scots pine (*Pinus sylvestris* L.) needles and WSCs in ECM sporocarps). Open circles indicate the correlation for *Russula* and *Lactarius*; open squares indicate the correlation for *Cortinarius*. For *Russula* and *Lactarius*, significant correlations with an adequate sample size (*n* > 3) are indicated with filled circles, denoting ‘effective numbers’ sufficient to filter out false positives due to autocorrelation in the VPD datasets. *Cortinarius* exhibited low effective numbers (*n* = 2) to filter out false positive significant time lags, suggesting no statistical significance between VPD signals and δ^13^C_WSC_ in *Cortinarius* can be determined. Hydrophobicity: Hi = hydrophilic*,* Ho = hydrophobic.

Pearson’s correlation analysis revealed distinct patterns in the relationship between δ^13^C_WSC_ of ECM sporocarps and time-integrated VPD (as a proxy for needle δ^13^C of sucrose) over a 30-day period. For each day in this period, we calculated the correlation coefficient between δ^13^C_WSC_ and VPD values from the preceding days, allowing us to determine the C transport time between Scots pine needles and ECM sporocarps (Figure 6). For the Hi genera *Russula* and *Lactarius*, correlation coefficients were significant between day 6 and day 13 (red circles, Pearson’s *r* > 0.80, *P* < 0.05), peaking at day 8 (Pearson’s *r* = 0.99), before gradually decreasing. This indicated that δ^13^C_WSC_ values in the Hi group showed the strongest correlation with time-integrated VPD values from 8 days prior to sporocarp sampling. These correlation patterns suggested that C fixed by the host tree takes ~6 to 13 days to be transported and incorporated into the WSCs of the Hi group.

For Ho genus *Cortinarius*, correlation coefficients between time-integrated VPD and δ^13^C_WSC_ during the initial 20 days predominantly ranged between 0.65 and 0.90, showing no clear trend and reaching a maximum at day 18 (Pearson’s *r* = 0.91). Despite these relatively high correlations, no statistically significant relationship was observed for *Cortinarius* after accounting for autocorrelation by calculating the effective sample size (*n* = 2). Nevertheless, the 18-day lag time showed the strongest correlation, suggesting this as the most likely C transport time from Scots pine needles to *Cortinarius*.

## Discussion

### Detection of C transport time from canopy to belowground components using natural abundance δ^13^C

#### Preservation of VPD signals in δ^13^C_sucrose_: implications for C allocation from needles to below ground components

Considering the coarser temporal resolution of root sampling and the likelihood that root sucrose reflected assimilates integrated over extended periods, the observed similarity in seasonal δ^13^C_sucrose_ variations and values between needles and roots (Figure 3a) suggested that the VPD signal recorded in leaf photosynthates (Figure 4) was, overall, transmitted and preserved in root sucrose. The main difference was a lower amplitude of seasonal variation in roots, possibly reflecting integration over longer timescales (Lehtonen et al. 2025). As shown by Rinne-Garmston et al. (2023) for Scots pine saplings and Lehtonen et al. (2025) for mature *P. abies*, intra-seasonal variation originating from leaf-level photosynthesis can be well preserved in the δ^13^C of root sugars and annual rings, respectively, despite the influence of post-photosynthetic processes (Gessler et al. 2007).

Our analysis revealed the strongest significant correlation (Pearson’s *r* = 0.85, *P* < 0.05) between δ^13^C_sucrose_ in needles and time-integrated VPD when applying a 4-day integration period (from day 0 to 3) with a 0.9 daily weight, compared with other time-integrated VPD methods (Figure 4). This weighting implied a daily sucrose turnover rate of ~10%, with about 72.9% of the original sucrose still present after the integration period (0.9^3^). Additionally, incorporating a fraction of the VPD signal from day 0 to day 3 more accurately reflected the composition of the needle sucrose pool (Figure 5), which includes both new and older sucrose formed within 4 days. These findings aligned with a previous study at the same site, where Tang et al. (2023) reported that δ^13^C_sucrose_ in *P. sylvestris* needles in 2018 correlated best with VPD, compared with RH and PAR, particularly when a 0.7–0.8 daily weight was applied over the previous 3–5 days. Similar δ^13^C–VPD relationships have been observed in Scots pine growing under semi-arid conditions (Klein et al. 2005), highlighting VPD as a key environmental driver of δ^13^C_sucrose_ variation in Scots pine needles across contrasting climates.

In addition, previous studies have found that δ^13^C signals from needle sucrose in mature trees were well-preserved in soil respiration (Trudell et al. 2004, Kaiser et al. 2015, Wang et al. 2021), indicating that photosynthetic C isotope signals can persist along belowground C pathways. In our study, δ^13^C_glucose_ values in roots closely matched the bulk δ^13^C_TOM_ values in hyphae (Figure 3a), supporting substantial C flow from plants via roots to hyphae to support the formation of new sporocarps. Although sucrose in roots is hydrolysed into equimolar concentrations of glucose and fructose, fungal hyphae exhibit a strong preference for glucose uptake with high efficiency and restrict fructose uptake when glucose concentrations are above 50 μm (Nehls et al. 2010). This close agreement between δ^13^C_TOM_ of hyphae and δ^13^C of its C source (δ^13^C_glucose_ in roots) was consistent with findings from Tang et al. (2022), who showed that bulk δ^13^C_TOM_ in new needles reflected the δ^13^C of recent photosynthates during the initial growth stage. Together, these results supported a mechanistic pathway in which the δ^13^C_sucrose_ signal, shaped by time-integrated VPD, is transmitted from needles through roots to ECM hyphae, reflecting environmental influences on C flow to ECM fungi. This preservation underscored the value of δ^13^C_sucrose_ as a tracer of whole-plant C allocation in response to environmental drivers.

Nevertheless, while VPD integrates a temperature signal and provides a useful proxy for δ^13^C_sucrose_ during the main growing season, it may not fully capture seasonal changes in post-photosynthetic fractionation or C transport dynamics, particularly during autumn when direct δ^13^C_sucrose_ measurements were unavailable. Still, our approach is supported by previous work showing that integrated seasonal proxies, such as solar radiation, can reliably estimate C transfer lag to ECM sporocarps (Hobbie et al. 2023).

#### Carbon transport time from canopy to ECM sporocarps

By incorporating ‘effective numbers’ to address autocorrelation in VPD datasets, we ensured robust and reliable C transport time estimates. Our approach yielded lag times of 6–13 days for *Lactarius* and *Russula* sporocarps. These values are consistent with a pulse-labelling study, which reported canopy-to-ECM C transport lag of ~6 days in *P. sylvestris* (Högberg et al. 2010). Moreover, our results aligned closely with recent findings from a 19.7 m tall *P. sylvestris* stand in northern Sweden, where a 7-day lag was reported based on correlations between δ^13^C_TOM_ of ECM sporocarps and solar radiation, a proxy of plant Gross Primary Production (GPP) (Hobbie et al. 2023). For *Cortinarius*, our estimated C transport time lag was 18 days, but unlike *Lactarius* and *Russula*, the statistical significance could not be confirmed for its correlation. However, a similar lag of 17–21 days was reported by Hobbie et al. (2024) in mature *P. abies* forests in Switzerland, based on correlations between ECM sporocarp δ^13^C_TOM_ and solar radiation. While their estimate reflected an average across 138 sporocarps from 16 genera, it placed our finding for *Cortinarius* within a comparable temporal range. The absence of a significant relationship in our dataset may be attributed to the greater variability in δ^13^C_WSC_ observed for *Cortinarius*, which showed no clear seasonal trend compared with *Russula* and *Lactarius* (Figure 3b). Even with sample sizes on 27 August and 10 September (*n* = 8, 9), *Cortinarius* exhibited larger SDs for δ^13^C_WSC_ (−25.2 ± 1.0‰) and δ^13^C_TOM_ (−25.1 ± 1.0‰) compared with *Russula* and *Lactarius* (Table S1 and Figure S2 available as Supplementary Data at *Tree Physiology* Online). This variability likely resulted from *Cortinarius*’s distinct physiological characteristics, their stronger proteolytic capacity for accessing organic nitrogen from deeper and more ^13^C-enriched soil layers than *Russula* and *Lactarius* (Agerer and Raidl 2004, Hobbie et al. 2014). While *Russula* and *Lactarius* primarily utilize C from photosynthates with some contribution from recent litter-derived C, the δ^13^C values in *Cortinarius* are influenced by both plant- and soil-derived C. This dual influence may mask clear temporal patterns in δ^13^C_WSC_ and weaken correlations with time-integrated VPD. Notably, Hobbie et al. (2023) suggested that Ho fungi, including *Cortinarius*, require a longer time to acquire plant-derived C for sporocarp production, exhibiting a 10-day delay in sporocarp production compared with Hi fungi such as *Russula* and *Lactarius*. Thereby, our results provided further context for the observed differences in C transport time among these fungal genera. These findings demonstrated that, with proper experimental design and sufficient climatic variability, natural abundance δ^13^C analysis combined with environmental proxies can be used to estimate the time lag between canopy photosynthesis and ECM sporocarp formation. This approach is particularly valuable in systems where pulse labelling is not feasible, thereby supporting Hypothesis 1. Although our data indicated a longer lag time for *Cortinarius*, we do not consider Hypothesis 2, that C transport time differs among fungal genera, to be statistically supported, due to the lack of a significant correlation.

Notably, in our study, CSIA of needle sugars enabled the direct measurements of δ^13^C_sucrose_, which captured a strong VPD signal in photosynthates. This allowed us to identify VPD as the strongest environmental driver for estimating C transport time to ECM sporocarps. While our compound-specific approach provides more physiologically precise insight into C source signals, such measurements remain technically demanding and are not widely accessible. Furthermore, transport lags inferred from natural abundance δ^13^C data typically represent C transfer integrated over the growing season, limiting their ability to resolve short-term dynamics such as peak photosynthetic activity in late July, which pulse-labelling can capture. The reliability of environmental proxies, such as VPD, may also decline during extended cloudy or rainy periods, potentially introducing bias. Despite these limitations, our approach demonstrated that, when combined with suitable environmental drivers, natural abundance δ^13^C measurements can offer valuable insights into seasonal C allocation and transport in ECM systems.

### Fungal genera’s role in δ^13^C_WSC_ of ECM sporocarps

The sampling date significantly influenced δ^13^C_WSC_ in sporocarps (*P* < 0.001; Table 2), likely reflecting seasonal variations in photosynthetic C assimilation by the host plants, which are linked to climate variables such as VPD (Tang et al. 2022) and PAR (Hobbie et al. 2023). The ANCOVA result showed that fungal genus was a marginally significant factor (*P* = 0.06) on δ^13^C_WSC_ in sporocarps. This finding contrasted with two key studies that found significant differences in δ^13^C among these genera. Chen et al. (2019) analysed 96 specimens across 11 fungal genera in Finland (September–October), while Hobbie et al. (2023) examined 248 specimens across 13 genera in Sweden (July–September). Both studies reported significantly lower δ^13^C_TOM_ values in *Cortinarius* compared with *Lactarius* and *Russula*. The discrepancy between our results and previous findings may be explained by the fungal sampling periods. *Cortinarius* typically exhibits higher C demands from host plants than *Lactarius* and *Russula*, generally resulting in lower δ^13^C_TOM_ values due to less selective C uptake from photosynthetic C (primary source) and soil-derived C (secondary source) and preferential transport of ^13^C-depleted sugars due to their longer mycorrhiza than *Lactarius* and *Russula*. However, in our study, *Cortinarius* emerged later (2 August, DOY 214) than *Lactarius* and *Russula*, coinciding with peak needle δ^13^C_sucrose_ values in late July. This sampling period suggested that *Cortinarius* may have incorporated more ^13^C-enriched C than typically observed. Furthermore, limited *Cortinarius* replication on certain dates (*n* = 1–2 for DOY 214 and 233, Table S1 available as Supplementary Data at *Tree Physiology* Online) likely reduced statistical power and resulted in marginal statistical significance. Notably, we analysed δ^13^C_WSC_ of ECM sporocarps, whereas previous studies focused on δ^13^C_TOM_. Since TOM includes lipids, proteins and chitin, variations in chemical composition may affect δ^13^C results (Hobbie et al. 2012, 2014; Chen et al. 2019; Hobbie et al. 2023). However, our results showed no significant differences between δ^13^C_WSC_ and δ^13^C_TOM_ in sporocarps (independent *t*-test: *P* > 0.05; Table 1). This suggests that δ^13^C_WSC_, like δ^13^C_TOM_, reflects fungal hydrophobicity—an important trait of fungal genera, as described by Hobbie et al. (2023). These observations support Hypothesis 3, indicating that ECM fungal genera influence δ^13^C_WSC_ values in their sporocarps.

The significant fungal genus and distance interaction (*P* = 0.02) indicated that the effect of distance varied by genus, suggesting that different ECM genera exhibit distinct spatial responses to host proximity, likely reflecting genus-specific strategies in C acquisition. These findings highlighted that δ^13^C variation in sporocarps cannot be explained by distance alone but depends on fungal identity. This interpretation is consistent with Hobbie et al. (2023), who reported that sporocarps closer to host trees (0–6 m) received substantially more recent photosynthates (71 ± 5%) than those at greater distances (6–12 m: 32 ± 6%; 12–18 m: 19 ± 7%).

### Ecological impacts of carbon transport time under warming

Deslippe et al. (2016) found that warming leads to a significant increase in high-biomass fungi with proteolytic capacity, particularly *Cortinarius* spp., while reducing fungi with high affinities for labile nitrogen, especially *Russula* spp., in Arctic tundra. These studies suggested that in response to global warming, *Cortinarius* may become more dominant in sensitive ECM fungal communities in the future, potentially altering belowground C allocation patterns (Hawkins et al. 2023). *Cortinarius* can enhance belowground C allocation due to high fungal biomass demand (Hobbie et al. 2012, Hobbie et al. 2023). The longer C transport time to its sporocarps observed in our study is consistent with its extensive mycelial network and exploration strategy, which may result in slower movement of recent photosynthates through the soil-fungal system (Hobbie et al. 2023b). However, their unique enzymatic degradation of complex organic matter may reduce long-term C retention in soil, as more C is cycled through fungal biomass and less becomes stabilized in humus (Tunlid et al. 2016). This trade-off could shift the fate of root-derived C and affect soil C stability. Moreover, Clemmensen et al. (2013) demonstrated that ECM fungi acted as a critical regulator of ecosystem C dynamics in boreal forests. If *Cortinarius* indeed has a longer C transport time compared with *Russula* and *Lactarius*, this could have ecological implications, including slower C cycling in soil ecosystems, reduced nutrient release from organic matter and decreased efficiency of C stabilization in soil (Clemmensen et al. 2021). These changes can reduce soil fertility, the function of the soil as a C sink and overall forest production.

## Conclusions

By using natural abundance δ^13^C and CSIA, this study provided a less invasive approach to estimating C transport time between Scots pine trees and ECM fungi. This method offered a viable alternative to the conventional ^13^C-pulse labelling technique and enhances our understanding of C dynamics and plant–fungal interactions in forest ecosystems, particularly under the influence of climate change. Although fungal genera had a marginally significant impact on δ^13^C_WSC_ of ECM sporocarps, the limited statistical power of our analysis prevented definitive conclusions. However, our findings suggested that fungal genera may play a role in influencing temporal C dynamics and allocation in ECM sporocarps. A time lag of 6 to 13 days was observed between C fixation in Scots pine and its allocation to *Russula* and *Lactarius* sporocarps, while *Cortinarius* exhibited the highest correlation of transport time at 18 days, though this statistical relationship cannot be determined. These findings highlighted the distinct C transport dynamics between *Cortinarius* versus *Russula* and *Lactarius*, potentially indicating different traits of C transfer in hydrophilic and hydrophobic fungal, which may have important implications for ecosystem C cycling. Given that ECM fungi significantly contribute to C reservoirs and C flux in soil, differences in transport times could affect C allocation and turnover rates in soil, with implications for nutrient cycling in soil, forest productivity and long-term C balance (Lindahl et al. 2021, Hawkins et al. 2023). This highlights the need for further research on the roles of different fungal genera in ecosystem-scale C dynamics. Such knowledge is essential for predicting and managing forest ecosystem responses to ongoing climate change.

## Supplementary Material

Supplement_File_R2_tpaf130

## Data Availability

The data that support the findings of this study are available from Envidatat WSL Birmensdorf.

## References

[ops-bib-reference-wmit7agqhp69mjiw] Aalto J, Aalto P, Keronen P et al. (2019) SMEAR II Hyytiälä forest meteorology, greenhouse gases, air quality and soil (Version 1). University of Helsinki, Institute for Atmospheric and Earth System Research. 10.23729/2001890a-2f0b-4e37-8c70-4d2cb5f40273.

[ref1] Aalto J, Aalto P, Keronen P et al. (2023) SMEAR II Hyytiälä forest meteorology, greenhouse gases, air quality and soil. University of Helsinki, Institute for Atmospheric and Earth System Research, Helsinki, Finland.

[ref2] Abazajian KN, Adelman-McCarthy JK, Agüeros MA et al. (2009) The seventh data release of the Sloan digital sky survey. Astrophys J Suppl Ser 182:543–558. 10.1088/0067-0049/182/2/543.

[ops-bib-reference-gmit6fuwl4aiglhq] Agerer R . (2001) Exploration types of ectomycorrhizae. Mycorrhiza 11(2), 107–114. 10.1007/s005720100108.

[ref3] Agerer R, Raidl S (2004) Distance-related semi-quantitative estimation of the extramatrical ectomycorrhizal mycelia of *Cortinarius obtusus* and *Tylospora asterophora*. Mycol Prog 3:57–64. 10.1007/s11557-006-0077-9.

[ref4] Averill C, Turner BL, Finzi AC (2014) Mycorrhiza-mediated competition between plants and decomposers drives soil carbon storage. Nature 505:543–545. 10.1038/nature12901. 24402225

[ref5] Bennett AE, Classen AT (2020) Climate change influences mycorrhizal fungal--plant interactions, but conclusions are limited by geographical study bias. Ecology 101:e02978. 10.1002/ecy.2978. 31953955

[ref7] Bowling DR, McDowell NG, Bond BJ, Law BE, Ehleringer JR (2002) ^13^C content of ecosystem respiration is linked to precipitation and vapor pressure deficit. Oecologia 131:113–124. 10.1007/s00442-001-0851-y. 28547501

[ref8] Bowling DR, Pataki DE, Randerson JT (2008) Carbon isotopes in terrestrial ecosystem pools and CO_2_ fluxes. New Phytol 178:24–40. 10.1111/j.1469-8137.2007.02342.x. 18179603

[ref9] Brendel O, Handley L, Griffiths H (2003) The δ^13^C of Scots pine (*Pinus sylvestris* L.) needles: spatial and temporal variations. Ann For Sci 60:97–104. 10.1051/forest:2003001.

[ref10] Brüggemann N, Gessler A, Kayler Z et al. (2011) Carbon allocation and carbon isotope fluxes in the plant-soil-atmosphere continuum: a review. Biogeosciences 8:3457–3489. 10.5194/bg-8-3457-2011.

[ref11] Chen J, Heikkinen J, Hobbie EA, Rinne-Garmston KT, Penttilä R, Mäkipää R (2019) Strategies of carbon and nitrogen acquisition by saprotrophic and ectomycorrhizal fungi in Finnish boreal *Picea abies*-dominated forests. Fungal Biol 123:456–464. 10.1016/j.funbio.2019.03.005. 31126422

[ref13] Clemmensen KE, Bahr A, Ovaskainen O et al. (2013) Roots and associated fungi drive long-term carbon sequestration in boreal forest. Science 340:1615–1618. 10.1126/science.1231923 23539604

[ops-bib-reference-ymitap5yqgj15mia] Clemmensen KE, Durling MB, Michelsen A, Hallin S, Finlay RD, Lindahl, BD. (2021) A tipping point in carbon storage when forest expands into tundra is related to mycorrhizal recycling of nitrogen. Ecology Letters 24(6):1193–1204. Portico. 10.1111/ele.13735. 33754469

[ref15] Colpaert JV, Van Tichelen KK (1996) Decomposition, nitrogen and phosphorus mineralization from beech leaf litter colonized by ectomycorrhizal or litter-decomposing basidiomycetes. New Phytol 134:123–132. 10.1111/j.1469-8137.1996.tb01152.x.

[ref16] Dahlberg A, Jonsson L, Nylund J-E (1997) Species diversity and distribution of biomass above and below ground among ectomycorrhizal fungi in an old-growth Norway spruce forest in South Sweden. Can J Bot 75:1323–1335. 10.1139/b97-844.

[ref17] Dannoura M, Maillard P, Fresneau C et al. (2011) *In situ* assessment of the velocity of carbon transfer by tracing ^13^C in trunk CO_2_ efflux after pulse labelling: variations among tree species and seasons. New Phytol 190:181–192. 10.1111/j.1469-8137.2010.03599.x. 21231935

[ref18] Dawdy DR, O’Donnell T, Prasad R (1964) Discussion of “nonlinear instantaneous unit-hydrograph theory”. J Hydraul Div 90:287–292. 10.1061/JYCEAJ.0001119.

[ref19] Deslippe JR, Hartmann M, Grayston SJ, Simard SW, Mohn WW (2016) Stable isotope probing implicates a species of *Cortinarius* in carbon transfer through ectomycorrhizal fungal mycelial networks in Arctic tundra. New Phytol 210:383–390. 10.1111/nph.13797. 26681156

[ref22] Ekblad A, Högberg P (2001) Natural abundance of ^13^C in CO_2_ respired from forest soils reveals speed of link between tree photosynthesis and root respiration. Oecologia 127:305–308. 10.1007/s004420100667. 28547099

[ref23] Ekblad A, Wallander H, Godbold DL et al. (2013) The production and turnover of extramatrical mycelium of ectomycorrhizal fungi in forest soils: role in carbon cycling. Plant Soil 366:1–27. 10.1007/s11104-013-1630-3.

[ref24] Epron D, Bahn M, Derrien D et al. (2012) Pulse-labelling trees to study carbon allocation dynamics: a review of methods, current knowledge and future prospects. Tree Physiol 32:776–798. 10.1093/treephys/tps057. 22700544

[ref25] Gessler A, Keitel C, Kodama N, Weston C, Winters AJ, Keith H, Grice K, Leuning R, Farquhar GD (2007) δ^13^C of organic matter transported from the leaves to the roots in *Eucalyptus delegatensis*: short-term variations and relation to respired CO_2_. Funct Plant Biol 34:692–706. 10.1071/FP07064. 32689397

[ref26] Hari P, Nikinmaa E, Pohja T, Siivola E, Bäck J, Vesala T, Kulmala M (2013) Station for measuring ecosystem-atmosphere relations: SMEAR. In Physical and Physiological Forest Ecology. 471–487. Dordrecht: Springer Netherlands. 10.1007/978-94-007-5603-8_9.

[ref27] Hawkins HJ, Cargill RI, Van Nuland ME, Hagen SC, Field KJ, Sheldrake M, Soudzilovskaia NA, Kiers ET (2023) Mycorrhizal mycelium as a global carbon pool. Curr Biol 33:R560–R573. 10.1016/j.cub.2023.02.027. 37279689

[ref29] Hobbie EA, Jocher G, Peichl M, Zhao P, Zhou Z, Hasselquist NJ (2023 *a*) Ectomycorrhizal hydrophobicity influences ectomycorrhizal C dynamics, N dynamics, and fruiting patterns in N addition experiments under pine. PREPRINT (Version 1) available at Research Square 10.21203/rs.3.rs-3657801/v1.

[ref33] Hobbie JE, Hobbie EA (2006) ^15^N in symbiotic fungi and plants estimates nitrogen and carbon flux rates in Arctic tundra. Ecology 87:816–822. 10.1890/0012-9658(2006)87[816:NISFAP]2.0.CO;2. 16676524

[ref31] Hobbie EA, Sánchez FS, Rygiewicz PT (2012) Controls of isotopic patterns in saprotrophic and ectomycorrhizal fungi. Soil Biol Biochem 48:60–68. 10.1016/j.soilbio.2012.01.014.

[ref28] Hobbie EA, Hofmockel KS, van Diepen LTA, Lilleskov EA, Ouimette AP, Finzi AC (2014) Fungal carbon sources in a pine forest: evidence from a ^13^C-labeled global change experiment. Fungal Ecol 10:91–100. 10.1016/j.funeco.2013.11.001. 24304469

[ref32] Hobbie EA, Siegwolf R, Körner C, Steinmann K, Wilhelm M, Saurer M, Keel SG (2023) Weather modifies the spatial extent of carbohydrate transfers from CO_2_-supplied broad-leaved trees to ectomycorrhizal fungi. Plant Soil 494:717–730. 10.1007/s11104-023-06314-x.

[ref30] Hobbie EA, Keel SG, Klein T, Rog I, Saurer M, Siegwolf R, Routhier MR, Körner C (2024) Tracing the spatial extent and lag time of carbon transfer from *Picea abies* to ectomycorrhizal fungi differing in host type, taxonomy, or hyphal development. Fungal Ecol 68:101315. 10.1016/j.funeco.2023.101315.

[ops-bib-reference-fmisyv901seeh3tc] Hobbie EA, Agerer R. (2010) Nitrogen isotopes in ectomycorrhizal sporocarps correspond to belowground exploration types. Plant and Soil 327(1):71–83. 10.1007/s11104-009-0032-z.

[ref34] Högberg MN, Briones MJI, Keel SG et al. (2010) Quantification of effects of season and nitrogen supply on tree below-ground carbon transfer to ectomycorrhizal fungi and other soil organisms in a boreal pine forest. New Phytol 187:485–493. 10.1111/j.1469-8137.2010.03274.x. 20456043

[ref36] Högberg P, Nordgren A, Buchmann N, Taylor AFS, Ekblad A, Högberg MN, Nyberg G, Ottosson-Löfvenius M, Read DJ (2001) Large-scale forest girdling shows that current photosynthesis drives soil respiration. Nature 411:789–792. 10.1038/35081058. 11459055

[ref35] Högberg P, Högberg MN, Göttlicher SG et al. (2008) High temporal resolution tracing of photosynthate carbon from the tree canopy to forest soil microorganisms. New Phytol 177:220–228. 10.1111/j.1469-8137.2007.02238.x. 17944822

[ref37] Huang J, Ladd SN, Ingrisch J et al. (2024) The mobilization and transport of newly fixed carbon are driven by plant water use in an experimental rainforest under drought. J Exp Bot 75:2545–2557. 10.1093/jxb/erae030. 38271585 PMC11358253

[ops-bib-reference-bmit8o5anloe2h5j] Kaiser C, Kilburn MR, Clode PL, Fuchslueger L, Koranda M, Cliff JB, Solaiman ZM, Murphy DV. (2014) Exploring the transfer of recent plant photosynthates to soil microbes: mycorrhizal pathway vs direct root exudation. New Phytologist 205(4):1537–1551. Portico. 10.1111/nph.13138. 25382456 PMC4357392

[ref38] Klein T, Hemming D, Lin T, Grünzweig JM, Maseyk K, Rotenberg E, Yakir D (2005) Association between tree-ring and needle δ^13^C and leaf gas exchange in *Pinus halepensis* under semi-arid conditions. Oecologia 144:45–54. 10.1007/s00442-005-0002-y. 15868163

[ref39] Klein T, Rog I, Livne-Luzon S, van der Heijden MG, Körner C (2023) Belowground carbon transfer across mycorrhizal networks among trees: facts, not fantasy. Open Res Europe 3:168. 10.12688/openreseurope.16594.1. PMC10751480 38152158

[ref40] Knohl A, Werner RA, Brand WA, Buchmann N (2005) Short-term variations in δ^13^C of ecosystem respiration reveals link between assimilation and respiration in a deciduous forest. Oecologia 142:70–82. 10.1007/s00442-004-1702-4. 15378343

[ref41] Kuzyakov Y, Gavrichkova O (2010) Time lag between photosynthesis and carbon dioxide efflux from soil: a review of mechanisms and controls. Glob Chang Biol 16:3386–3406. 10.1111/j.1365-2486.2010.02179.x.

[ref42] Lehtonen A, Heikkinen J, Boroski CA et al. (2025) Carbon allocation to roots of suppressed Norway spruce increases immediately after selection harvest. For Ecol Manage 585:122645. 10.1016/j.foreco.2025.122645.

[ref43] Leppä K, Tang Y, Ogée J et al. (2022) Explicitly accounting for needle sugar pool size crucial for predicting intra-seasonal dynamics of needle carbohydrates δ^18^O and δ^13^C. New Phytol 236:2044–2060. 35575976 10.1111/nph.18227 PMC9795997

[ref44] Lilleskov EA, Hobbie EA, Horton TR (2011) Conservation of ectomycorrhizal fungi: exploring the linkages between functional and taxonomic responses to anthropogenic N deposition. Fungal Ecol 4:174–183. 10.1016/j.funeco.2010.09.008.

[ref45] Lindahl BD, Kyaschenko J, Varenius K, Clemmensen KE, Dahlberg A, Karltun E, Stendahl J (2021) A group of ectomycorrhizal fungi restricts organic matter accumulation in boreal forest. Ecol Lett 24:1341–1351. 10.1111/ele.13746. 33934481

[ref46] Machacova K, Vainio E, Urban O, Pihlatie M (2019) Seasonal dynamics of stem N_2_O exchange follow the physiological activity of boreal trees. Nat Commun 10:1–13. 10.1038/s41467-019-12976-y. 31676776 PMC6825224

[ref47] Martin F, Aerts A, Ahrén D et al. (2008) The genome of *Laccaria bicolor* provides insights into mycorrhizal symbiosis. Nature 452:88–92. 10.1038/nature06556. 18322534

[ref48] McDowell NG, Bowling DR, Bond BJ, Irvine J, Law BE, Anthoni P, Ehleringer JR (2004) Response of the carbon isotopic content of ecosystem, leaf, and soil respiration to meteorological and physiological driving factors in a *Pinus ponderosa* ecosystem. Global Biogeochem Cycles 18:1013. 10.1029/2003GB002049.

[ref50] Moyano FE, Kutsch WL, Rebmann C (2008) Soil respiration fluxes in relation to photosynthetic activity in broad-leaf and needle-leaf forest stands. Agric For Meteorol 148:135–143. 10.1016/j.agrformet.2007.09.006.

[ref51] Nara K, Nakaya H, Hogetsu T (2003) Ectomycorrhizal sporocarp succession and production during early primary succession on Mount Fuji. New Phytol 158:193–206. 10.1046/j.1469-8137.2003.00724.x. 33873602

[ref53] Nehls U, Hampp R (2000) Carbon allocation in ectomycorrhizas. Physiol Mol Plant Pathol 57:95–100. 10.1094/MPMI.1998.11.3.167 9487692

[ref52] Nehls U, Göhringer F, Wittulsky S, Dietz S (2010) Fungal carbohydrate support in the ectomycorrhizal symbiosis: a review. Plant Biol 12:292–301. 10.1111/j.1438-8677.2009.00312.x. 20398236

[ref55] Phillips CL, Kluber LA, Martin JP, Caldwell BA, Bond BJ (2012) Contributions of ectomycorrhizal fungal mats to forest soil respiration. Biogeosciences 9:2099–2110. 10.5194/bg-9-2099-2012.

[ref56] Pickles BJ, Egger KN, Massicotte HB, Green DS (2012) Ectomycorrhizas and climate change. Fungal Ecol 5:73–84. 10.1016/j.funeco.2011.08.009.

[ref57] Pirinen P, Simola H, Aalto J, Kaukoranta JP, Karlsson P, Ruuhela R (2012) Climatological statistics of Finland 1981--2010. Finnish Meteorol Inst Rep 1:1–96.

[ref58] Rapaport A, Livne-Luzon S, Fox H, Oppenheimer-Shaanan Y, Klein T (2024) Rapid and chemically diverse C transfer from trees to mycorrhizal fruit bodies in the forest. Funct Ecol 39:1343–1357. 10.1111/1365-2435.14541.

[ref59] Read DJ, Perez-Moreno J (2003) Mycorrhizas and nutrient cycling in ecosystems--a journey towards relevance? New Phytol 157:475–492. 10.1046/j.1469-8137.2003.00704.x. 33873410

[ref60] Ren R, Yue X, Li J, Xie S, Guo S, Zhang Z (2020) Coexpression of sucrose synthase and the SWEET transporter, which are associated with sugar hydrolysis and transport, respectively, increases the hexose content in *Vitis vinifera* L. grape berries. Front Plant Sci 11:321. 10.3389/fpls.2020.00321. 32457764 PMC7221319

[ref61] Rinne KT, Saurer M, Kirdyanov AV, Bryukhanova MV, Prokushkin AS, Churakova Sidorova OV, Siegwolf RTW (2015) Examining the response of needle carbohydrates from Siberian larch trees to climate using compound-specific δ^13^C and concentration analyses. Plant Cell Environ 38:2340–2352. 10.1111/pce.12554. 25916312

[ref62] Rinne KT, Saurer M, Streit K, Siegwolf RTW (2012) Evaluation of a liquid chromatography method for compound-specific δ^13^C analysis of plant carbohydrates in alkaline media. Rapid Commun Mass Spectrom 26:2173–2185. 10.1002/rcm.6334. 22886814

[ref63] Rinne-Garmston KT, Tang Y, Sahlstedt E et al. (2023) Drivers of intra-seasonal δ^13^C signal in tree-rings of *Pinus sylvestris* as indicated by compound-specific and laser ablation isotope analysis. Plant Cell Environ 46:2649–2666. 10.1111/pce.14636. 37312624

[ref65] Streit K, Rinne KT, Hagedorn F, Dawes MA, Saurer M, Hoch G, Werner RA, Buchmann N, Siegwolf RTW (2012) Tracing fresh assimilates through *Larix decidua* exposed to elevated CO_2_ and soil warming at the alpine treeline using compound-specific stable isotope analysis. New Phytol 197:838–849. 23252478 10.1111/nph.12074

[ops-bib-reference-umit6jyrgbbbohpi] Suz LM, Barsoum N, Benham S et al. (2014) Environmental drivers of ectomycorrhizal communities in Europe’s temperate oak forests. Molecular Ecology 23(22):5628–5644. Portico. 10.1111/mec.12947. 25277863

[ref66] Tang Y, Schiestl-Aalto P, Lehmann MM, Saurer M, Sahlstedt E, Kolari P, Leppä K, Bäck J, Rinne-Garmston KT (2023) Estimating intra-seasonal photosynthetic discrimination and water use efficiency using δ^13^C of leaf sucrose in Scots pine. J Exp Bot 74:321–335. 36255219 10.1093/jxb/erac413 PMC9786842

[ref67] Tang Y, Schiestl-Aalto P, Saurer M et al. (2022) Tree organ growth and carbon allocation dynamics impact the magnitude and δ^13^C signal of stem and soil CO_2_ fluxes. Tree Physiol 42:2404–2418. 35849053 10.1093/treephys/tpac079 PMC10101690

[ops-bib-reference-cmit8iindu23hcqe] Trudell SA, Rygiewicz PT, Edmonds RL. (2004) Patterns of nitrogen and carbon stable isotope ratios in macrofungi, plants and soils in two old‐growth conifer forests. New Phytologist 164(2):317–335. Portico. 10.1111/j.1469-8137.2004.01162.x. 33873563

[ops-bib-reference-kmit96de21v58gjt] Tunlid A, Floudas D, Koide R, Rineau F. (2016) Soil organic matter decomposition mechanisms in ectomycorrhizal fungi. Molecular mycorrhizal symbiosis. 257–275. 10.1002/9781118951446.ch15.

[ref69] Vogt PR, Taylor PT, Kovacs LC, Johnson GL (1982) The Canada Basin: aeromagnetic constraints on structure and evolution. Tectonophysics 89:295–336.

[ref70] Wanek W, Heintel S, Richter A (2001) Preparation of starch and other carbon fractions from higher plant leaves for stable carbon isotope analysis. Rapid Commun Mass Spectrom 15:1136–1140. 11445894 10.1002/rcm.353

[ref71] Wang R, Bicharanloo B, Shirvan MB, Cavagnaro TR, Jiang Y, Keitel C, Dijkstra FA (2021) A novel ^13^C pulse-labelling method to quantify the contribution of rhizodeposits to soil respiration in a grassland exposed to drought and nitrogen addition. New Phytol 230:857–866. 33253439 10.1111/nph.17118

[ref72] Wingate L, Ogée J, Burlett R, Bosc A, Devaux M, Grace J, Loustau D, Gessler A (2010) Photosynthetic carbon isotope discrimination and its relationship to the carbon isotope signals of stem, soil and ecosystem respiration. New Phytol 188:576–589. 20663061 10.1111/j.1469-8137.2010.03384.x

