# Supplementary material for: Determining the carbon transport time from Scots pine (Pinus sylvestris L.) needles to ectomycorrhizal sporocarps using the natural abundance carbon isotopic composition
Source: Tree Physiol. 2025 Oct 22;45(12):tpaf130. doi: 10.1093/treephys/tpaf130 (PMC12716283; doi:10.1093/treephys/tpaf130)
Supplement: Supplement_File_R2_tpaf130 [file supplement_file_r2_tpaf130.docx]

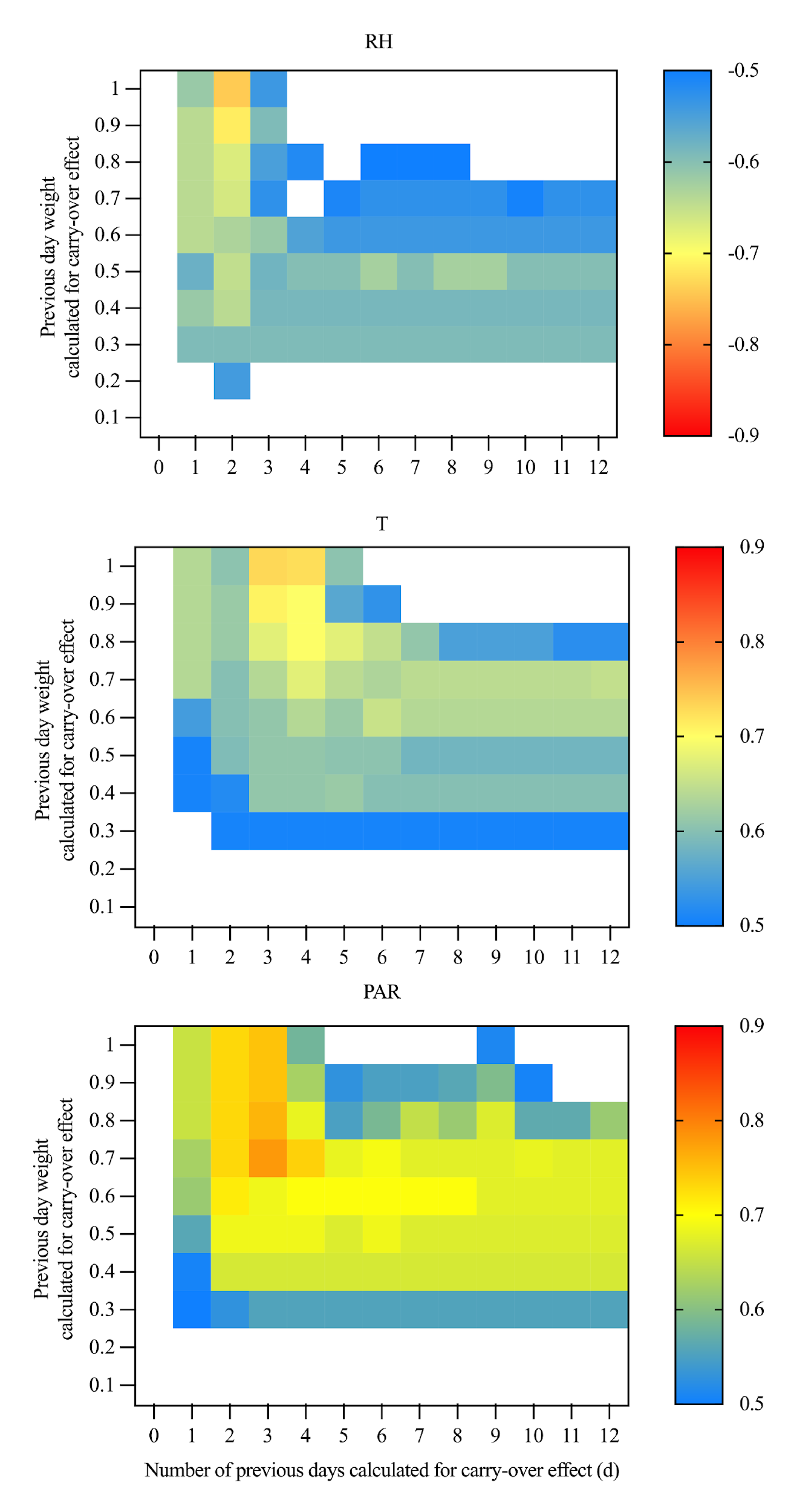


**Supplement Fig. 1** Environmental signals imprinted in δ^13^C of needle sucrose of pine (*Pinus sylvestris*) at Hyytiälä during the growing season of 2019. The analyzed variables, including δ^13^C of sucrose of current-year needles, photosynthetically active radiation (PAR), relative humidity (RH), and air temperature (T) were integrated with a carry-over effect and a varying previous day weight (Eq. 4). *Pearson*’s correlation coefficient (r) is indicated by the colors from blue (low values) to red (high values). Only significant results (r > 0.5, p < 0.05) are presented.


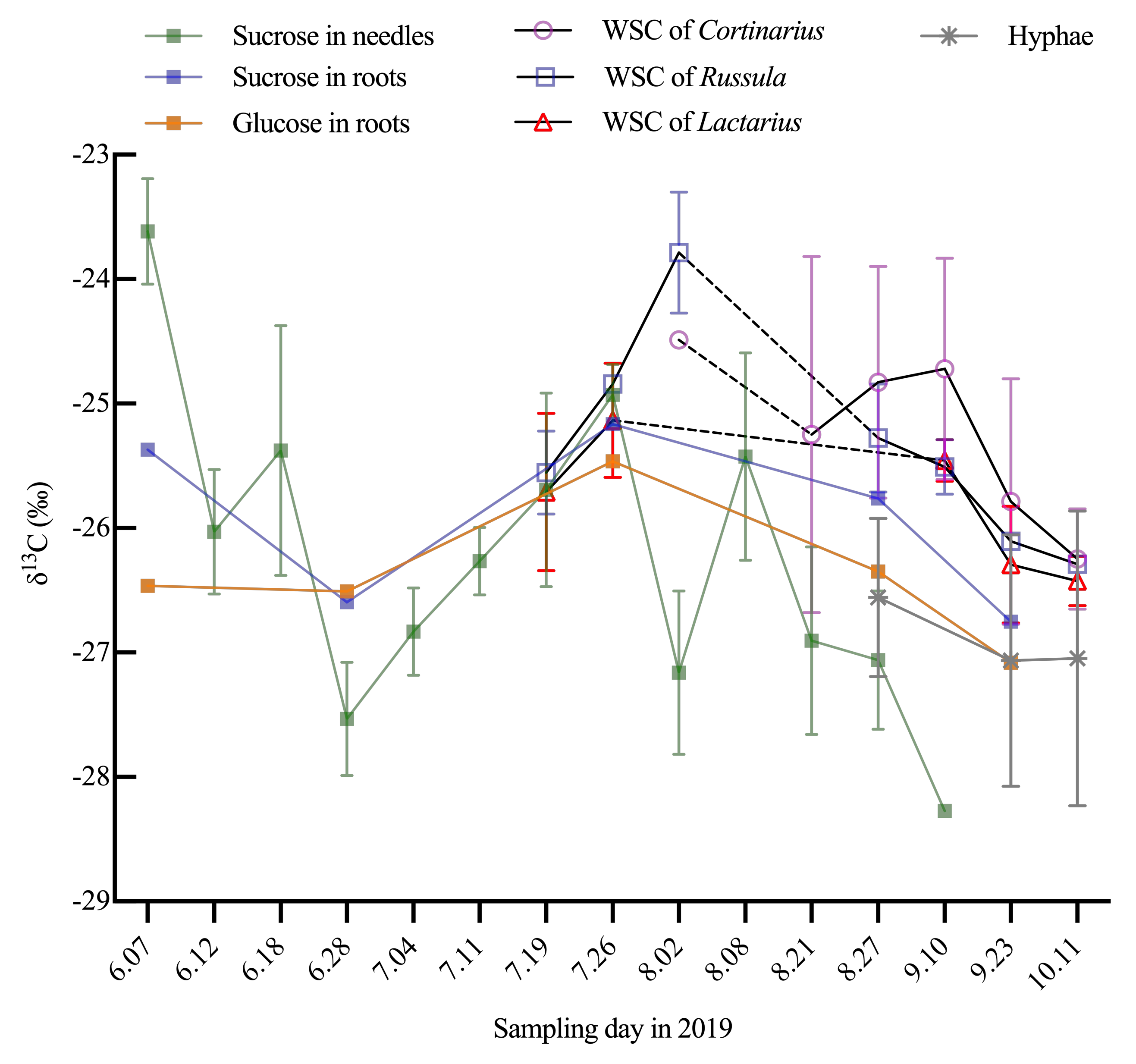


**Supplement Fig. 2** Comparison of intra-seasonal δ^13^C of sugar (i.e. sucrose, glucose) dynamics in pine (*Pinus sylvestris*) tree tissues, water-soluble carbohydrates (WSC) in sporocarps of three ectomycorrhizal species, and total organic matter of hyphae. Mean values and standard deviation are shown (root were pooled; needles (n)= 5; hyphae (n)=6-13; sporocarps: *Cortinarius* (n) = 1-9, *Russula* (n) = 1-5, *Lactarius* (n) = 3-14)

**Supplement Table. 1** Numbers of sporocarps and hyphae of three ectomycorrhizal species at their sampling dates (date of year) during the fungal collection period between 19 July and 11 October 2019. ‘-’ represents no fungi collected. Hydrophobicity: Hi = hydrophilic*,* Ho = hydrophobic.

| Sampling dates (DOY) | Fungi species (numbers) | | | | Hyphae |
| --- | --- | --- | --- | --- | --- |
|  | *Lactarius* (33) | *Russula* (15) | Hi group (48) | *Cortinarius*  Ho fungi (30) |  |
| 7.19 (200) | 6 | 5 | 11 | - | - |
| 7.26 (207) | 6 | 1 | 7 | - | - |
| 8.02 (214) | - | 2 | 2 | 1 | - |
| 8.21 (233) | - | - | - | 2 | - |
| 8.27 (239) | - | 3 | 3 | 8 | - |
| 9.10 (253) | 3 | 2 | 5 | 9 | 6 |
| 9.23 (266) | 14 | 1 | 15 | 5 | 13 |
| 10.11 (284) | 4 | 1 | 5 | 5 | 12 |
